# *N*,*N*′-Di-8-quinolyladipamide

**DOI:** 10.1107/S1600536809017541

**Published:** 2009-05-23

**Authors:** Shi-Ying Wang, Xing-Lei Xie, Qing-Hua Huang, Yu-Sheng Lin

**Affiliations:** a College of Chemistry and Molecular Engineering, Qingdao University of Science and Technology, Qingdao 266042, People’s Republic of China

## Abstract

The complete molecule of the title compound, C_24_H_22_N_4_O_2_, is generated by a crystallographic inversion centre located at the mid-point of the central C—C bond. The quinoline ring system and the hexyl chain are both essentially planar, and the dihedral angle between them is 46.30 (2)°. Intra­molecular N—H⋯N and C—H⋯O hydrogen bonds form five- and six-numbered rings, respectively. The crystal packing is stabilized by short C—H⋯O inter­actions.

## Related literature

For details of the synthesis, see: Chen *et al.* (2007 ▶). For related structures, see: Chen *et al.* (2007 ▶); Wen *et al.* (2006 ▶).
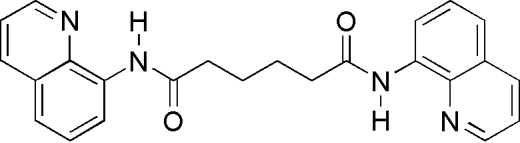


## Experimental

### 

#### Crystal data

C_24_H_22_N_4_O_2_
*M* *_r_* = 398.46
Monoclinic, 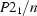

*a* = 9.923 (2) Å
*b* = 9.184 (2) Å
*c* = 11.722 (3) Å
β = 110.530 (4)°
*V* = 1000.4 (4) Å^3^
*Z* = 2
Mo *K*α radiation
μ = 0.09 mm^−1^
*T* = 294 K
0.24 × 0.20 × 0.12 mm

#### Data collection

Bruker SMART CCD area-detector diffractometer
Absorption correction: multi-scan (*SADABS*; Sheldrick,1996 ▶) *T*_min_ = 0.980, *T*_max_ = 0.990
5622 measured reflections
2048 independent reflections
1274 reflections with *I* > 2σ(*I*)
*R*_int_ = 0.032

#### Refinement

*R*[*F* ^2^ > 2σ(*F* ^2^)] = 0.042
*wR*(*F* ^2^) = 0.120
*S* = 1.00
2048 reflections
140 parameters
1 restraint
H atoms treated by a mixture of independent and constrained refinement
Δρ_max_ = 0.14 e Å^−3^
Δρ_min_ = −0.17 e Å^−3^

### 

Data collection: *SMART* (Bruker 2001 ▶); cell refinement: *SAINT* (Bruker 2001 ▶); data reduction: *SAINT*; program(s) used to solve structure: *SHELXTL* (Sheldrick, 2008 ▶); program(s) used to refine structure: *SHELXTL*; molecular graphics: *SHELXTL*; software used to prepare material for publication: *SHELXTL*.

## Supplementary Material

Crystal structure: contains datablocks I, global. DOI: 10.1107/S1600536809017541/fl2250sup1.cif

Structure factors: contains datablocks I. DOI: 10.1107/S1600536809017541/fl2250Isup2.hkl

Additional supplementary materials:  crystallographic information; 3D view; checkCIF report

## Figures and Tables

**Table 1:** Hydrogen-bond geometry (Å, °)

*D*—H⋯*A*	*D*—H	H⋯*A*	*D*⋯*A*	*D*—H⋯*A*
N2—H2*A*⋯N1	0.892 (9)	2.23 (2)	2.676 (2)	110.4 (15)
C7—H7⋯O1	0.93	2.33	2.902 (2)	119
C11—H11*B*⋯O1^i^	0.97	2.66	3.134 (2)	111

Symmetry code: (i) 
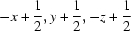
.

**Fig. 1.**
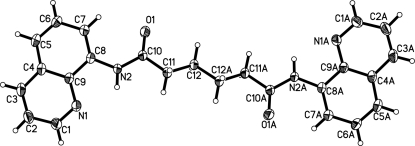
The molecular structure of (I), with atom labels and 30% probability displacement ellipsoids.

**Fig. 2.**
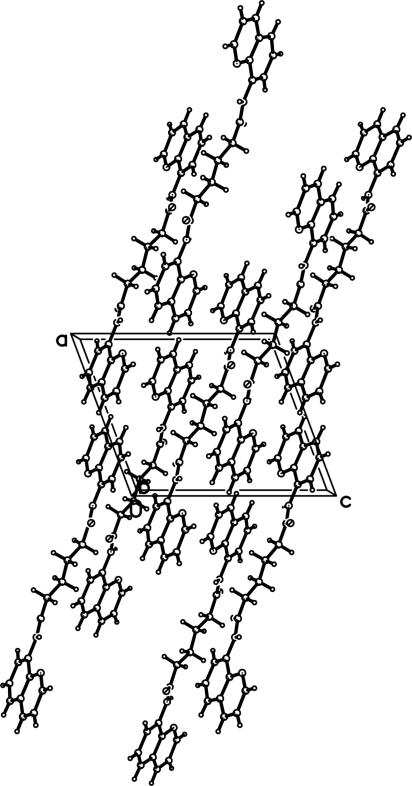
The packing diagram of (I), viewed down the b axis.

## supplementary crystallographic information

### Comment

Recently, Chen *et al.* (2007) reported the syntheses and crystal structures of the flexible ligand *N*,*N'*-di(2-pyridyl)adipamide and its several Ag(I) complexes. Theses complexes form topologically promising zigzag, helical or sinusoidal chain architectures because the flexible ligand can adopt three different conformations. To investigate the influence of the terminal groups on crystal structure, and to obtain a more topologically promising coordination framework, we synthesized and carried out the structure determination of the title compound, (I) (Fig. 1).

The molecule sits on a center of symmetry passing through the central C12—C12^ii^ bond [symmetry code: (ii): -*x*, -*y*, -*z*] (Fig. 1). All bond lengths and angles in (I) show normal values and are comparable to those of the related compounds, *N*,*N'*-di(2-pyridyl)adipamide (Chen *et al.*, 2007), and *N*-(quinolin-8-yl)-2-(quinolin-8-yloxy)acetamide (Wen *et al.*, 2006). The quinoline group is essentially planar, with a dihedral angle of 1.70 (3)° between the benzene ring (C4—C9) and pyridine ring (C1—C4/C9/N1). The C10—C12/C10A—C12A unit is also planar,  with the dihedral angle to the quinoline system of 46.30 (2)°. Two intramolecular hydrogen bonds, *viz.* N2—H2A···N1 and C7—H7···O1 (Fig. 1 and Table 1), form  five- and  six-membered rings, respectively, and affect the conformation of the molecule. The crystal packing is stabilized by short C11—H11B···O1  interactions (Fig. 2 and Table 1).

### Experimental

The title compound was synthesized by a reaction of adipoyl chloride and 8-aminoquinoline according to literature method (Chen *et al.*, 2007). Colourless single cystals suitable for X-ray diffraction were obtained by slow evaporation from a methanol solution over a period of 7 d.

### Refinement

H atoms were positioned geometrically, with N—H = 0.86 Å and C—H = 0.95–0.99 Å, respectively, and constrained to ride on their parent atoms, with *U*_iso_(H) = 1.2 *U*_eq_(C,*N*).

### Crystal data

**Table:**

C_24_H_22_N_4_O_2_	*F*(000) = 420
*M**_r_* = 398.46	*D*_x_ = 1.323 Mg m^−^^3^
Monoclinic, *P*2_1_/*n*	Mo *K*α radiation, λ = 0.71073 Å
Hall symbol: -P 2yn	Cell parameters from 1501 reflections
*a* = 9.923 (2) Å	θ = 2.9–25.3°
*b* = 9.184 (2) Å	µ = 0.09 mm^−^^1^
*c* = 11.722 (3) Å	*T* = 294 K
β = 110.530 (4)°	Column, colourless
*V* = 1000.4 (4) Å^3^	0.24 × 0.20 × 0.12 mm
*Z* = 2	

### Data collection

**Table:**

Bruker SMART CCD area-detector diffractometer	2048 independent reflections
Radiation source: fine-focus sealed tube	1274 reflections with *I* > 2σ(*I*)
graphite	*R*_int_ = 0.032
φ and ω scans	θ_max_ = 26.4°, θ_min_ = 2.3°
Absorption correction: multi-scan (*SADABS*; Sheldrick,1996)	*h* = −11→12
*T*_min_ = 0.980, *T*_max_ = 0.990	*k* = −11→9
5622 measured reflections	*l* = −9→14

### Refinement

**Table:**

Refinement on *F*^2^	Primary atom site location: structure-invariant direct methods
Least-squares matrix: full	Secondary atom site location: difference Fourier map
*R*[*F*^2^ > 2σ(*F*^2^)] = 0.042	Hydrogen site location: inferred from neighbouring sites
*wR*(*F*^2^) = 0.120	H atoms treated by a mixture of independent and constrained refinement
*S* = 1.00	*w* = 1/[σ^2^(*F*_o_^2^) + (0.0579*P*)^2^ + 0.1339*P*] where *P* = (*F*_o_^2^ + 2*F*_c_^2^)/3
2048 reflections	(Δ/σ)_max_ < 0.001
140 parameters	Δρ_max_ = 0.14 e Å^−^^3^
1 restraint	Δρ_min_ = −0.17 e Å^−^^3^

### Special details

**Table:**

Geometry. All e.s.d.'s (except the e.s.d. in the dihedral angle between two l.s. planes) are estimated using the full covariance matrix. The cell e.s.d.'s are taken into account individually in the estimation of e.s.d.'s in distances, angles and torsion angles; correlations between e.s.d.'s in cell parameters are only used when they are defined by crystal symmetry. An approximate (isotropic) treatment of cell e.s.d.'s is used for estimating e.s.d.'s involving l.s. planes.
Refinement. Refinement of *F*^2^ against ALL reflections. The weighted *R*-factor *wR* and goodness of fit *S* are based on *F*^2^, conventional *R*-factors *R* are based on *F*, with *F* set to zero for negative *F*^2^. The threshold expression of *F*^2^ > σ(*F*^2^) is used only for calculating *R*-factors(gt) *etc*. and is not relevant to the choice of reflections for refinement. *R*-factors based on *F*^2^ are statistically about twice as large as those based on *F*, and *R*- factors based on ALL data will be even larger.

### Fractional atomic coordinates and isotropic or equivalent isotropic displacement parameters (Å^2^)

**Table:**

	*x*	*y*	*z*	*U*_iso_*/*U*_eq_	
O1	0.31418 (15)	−0.13777 (14)	0.23248 (14)	0.0793 (5)	
N1	0.61637 (15)	0.28926 (17)	0.28795 (13)	0.0498 (4)	
N2	0.41475 (15)	0.08554 (16)	0.26119 (14)	0.0440 (4)	
C1	0.7142 (2)	0.3904 (2)	0.30187 (19)	0.0626 (6)	
H1	0.6886	0.4723	0.2522	0.075*	
C2	0.8543 (2)	0.3835 (3)	0.38633 (19)	0.0649 (6)	
H2	0.9192	0.4585	0.3917	0.078*	
C3	0.8942 (2)	0.2662 (2)	0.46005 (17)	0.0559 (5)	
H3	0.9869	0.2602	0.5172	0.067*	
C4	0.79507 (17)	0.1532 (2)	0.45011 (15)	0.0438 (5)	
C5	0.82757 (19)	0.0257 (2)	0.52156 (17)	0.0518 (5)	
H5	0.9178	0.0148	0.5816	0.062*	
C6	0.72814 (19)	−0.0807 (2)	0.50313 (17)	0.0529 (5)	
H6	0.7518	−0.1654	0.5494	0.063*	
C7	0.59014 (19)	−0.0659 (2)	0.41559 (16)	0.0475 (5)	
H7	0.5236	−0.1407	0.4044	0.057*	
C8	0.55222 (17)	0.05739 (19)	0.34647 (15)	0.0390 (4)	
C9	0.65611 (17)	0.16955 (18)	0.36128 (14)	0.0391 (4)	
C10	0.30407 (18)	−0.0079 (2)	0.21278 (16)	0.0455 (5)	
C11	0.16598 (18)	0.06244 (19)	0.13495 (17)	0.0477 (5)	
H11A	0.1883	0.1489	0.0975	0.057*	
H11B	0.1132	0.0930	0.1866	0.057*	
C12	0.07120 (17)	−0.03564 (19)	0.03641 (16)	0.0447 (5)	
H12A	0.0526	−0.1245	0.0731	0.054*	
H12B	0.1214	−0.0618	−0.0183	0.054*	
H2A	0.4032 (19)	0.1789 (11)	0.2388 (17)	0.057 (6)*	

### Atomic displacement parameters (Å^2^)

**Table:**

	*U*^11^	*U*^22^	*U*^33^	*U*^12^	*U*^13^	*U*^23^
O1	0.0648 (10)	0.0370 (8)	0.0939 (12)	−0.0048 (7)	−0.0250 (8)	0.0065 (8)
N1	0.0425 (9)	0.0574 (10)	0.0425 (9)	−0.0107 (8)	0.0061 (7)	0.0051 (8)
N2	0.0328 (8)	0.0376 (9)	0.0489 (9)	−0.0033 (6)	−0.0017 (7)	0.0043 (7)
C1	0.0590 (13)	0.0706 (14)	0.0506 (12)	−0.0212 (11)	0.0096 (10)	0.0115 (10)
C2	0.0532 (13)	0.0802 (15)	0.0543 (12)	−0.0290 (11)	0.0100 (10)	−0.0005 (11)
C3	0.0382 (10)	0.0782 (14)	0.0444 (11)	−0.0126 (10)	0.0058 (8)	−0.0063 (11)
C4	0.0344 (10)	0.0587 (12)	0.0354 (9)	−0.0033 (8)	0.0087 (8)	−0.0069 (9)
C5	0.0343 (10)	0.0684 (13)	0.0427 (10)	0.0058 (9)	0.0012 (8)	0.0007 (10)
C6	0.0446 (11)	0.0561 (12)	0.0479 (11)	0.0067 (9)	0.0037 (9)	0.0073 (9)
C7	0.0396 (10)	0.0478 (11)	0.0475 (11)	−0.0017 (8)	0.0060 (9)	0.0023 (9)
C8	0.0315 (9)	0.0452 (10)	0.0363 (9)	−0.0001 (7)	0.0068 (7)	−0.0019 (8)
C9	0.0347 (9)	0.0476 (10)	0.0334 (9)	−0.0005 (8)	0.0099 (8)	−0.0023 (8)
C10	0.0393 (10)	0.0374 (10)	0.0478 (10)	−0.0042 (8)	0.0005 (8)	−0.0004 (8)
C11	0.0379 (10)	0.0404 (10)	0.0521 (11)	−0.0023 (8)	0.0000 (9)	−0.0006 (9)
C12	0.0333 (10)	0.0419 (10)	0.0485 (10)	−0.0030 (7)	0.0014 (8)	0.0006 (8)

### Geometric parameters (Å, °)

**Table:**

O1—C10	1.213 (2)	C5—C6	1.351 (3)
N1—C1	1.312 (2)	C5—H5	0.9300
N1—C9	1.366 (2)	C6—C7	1.400 (2)
N2—C10	1.352 (2)	C6—H6	0.9300
N2—C8	1.404 (2)	C7—C8	1.366 (2)
N2—H2A	0.892 (9)	C7—H7	0.9300
C1—C2	1.397 (3)	C8—C9	1.424 (2)
C1—H1	0.9300	C10—C11	1.500 (2)
C2—C3	1.350 (3)	C11—C12	1.506 (2)
C2—H2	0.9300	C11—H11A	0.9700
C3—C4	1.407 (2)	C11—H11B	0.9700
C3—H3	0.9300	C12—C12^i^	1.519 (3)
C4—C5	1.410 (3)	C12—H12A	0.9700
C4—C9	1.415 (2)	C12—H12B	0.9700
			
C1—N1—C9	117.01 (16)	C8—C7—H7	119.7
C10—N2—C8	128.73 (15)	C6—C7—H7	119.7
C10—N2—H2A	119.0 (12)	C7—C8—N2	124.88 (16)
C8—N2—H2A	112.2 (12)	C7—C8—C9	119.34 (15)
N1—C1—C2	124.37 (19)	N2—C8—C9	115.76 (15)
N1—C1—H1	117.8	N1—C9—C4	122.70 (15)
C2—C1—H1	117.8	N1—C9—C8	117.95 (15)
C3—C2—C1	119.07 (18)	C4—C9—C8	119.36 (15)
C3—C2—H2	120.5	O1—C10—N2	122.93 (16)
C1—C2—H2	120.5	O1—C10—C11	122.42 (15)
C2—C3—C4	119.71 (18)	N2—C10—C11	114.63 (15)
C2—C3—H3	120.1	C10—C11—C12	113.62 (15)
C4—C3—H3	120.1	C10—C11—H11A	108.8
C3—C4—C5	123.78 (17)	C12—C11—H11A	108.8
C3—C4—C9	117.12 (17)	C10—C11—H11B	108.8
C5—C4—C9	119.08 (16)	C12—C11—H11B	108.8
C6—C5—C4	120.26 (17)	H11A—C11—H11B	107.7
C6—C5—H5	119.9	C11—C12—C12^i^	112.39 (18)
C4—C5—H5	119.9	C11—C12—H12A	109.1
C5—C6—C7	121.21 (17)	C12^i^—C12—H12A	109.1
C5—C6—H6	119.4	C11—C12—H12B	109.1
C7—C6—H6	119.4	C12^i^—C12—H12B	109.1
C8—C7—C6	120.70 (17)	H12A—C12—H12B	107.9
			
C9—N1—C1—C2	−0.3 (3)	C1—N1—C9—C8	−179.10 (17)
N1—C1—C2—C3	−0.3 (3)	C3—C4—C9—N1	−0.7 (2)
C1—C2—C3—C4	0.5 (3)	C5—C4—C9—N1	−179.61 (16)
C2—C3—C4—C5	178.89 (19)	C3—C4—C9—C8	179.22 (16)
C2—C3—C4—C9	0.0 (3)	C5—C4—C9—C8	0.3 (2)
C3—C4—C5—C6	−177.26 (18)	C7—C8—C9—N1	177.82 (16)
C9—C4—C5—C6	1.6 (3)	N2—C8—C9—N1	−3.5 (2)
C4—C5—C6—C7	−1.7 (3)	C7—C8—C9—C4	−2.1 (2)
C5—C6—C7—C8	−0.2 (3)	N2—C8—C9—C4	176.61 (15)
C6—C7—C8—N2	−176.52 (17)	C8—N2—C10—O1	−5.4 (3)
C6—C7—C8—C9	2.1 (3)	C8—N2—C10—C11	173.11 (17)
C10—N2—C8—C7	−14.2 (3)	O1—C10—C11—C12	−30.0 (3)
C10—N2—C8—C9	167.19 (17)	N2—C10—C11—C12	151.44 (17)
C1—N1—C9—C4	0.8 (3)	C10—C11—C12—C12^i^	176.88 (18)

Symmetry codes: (i) −*x*, −*y*, −*z*.

### Hydrogen-bond geometry (Å, °)

**Table:**

*D*—H···*A*	*D*—H	H···*A*	*D*···*A*	*D*—H···*A*
N2—H2A···N1	0.89 (1)	2.23 (2)	2.676 (2)	110 (2)
C7—H7···O1	0.93	2.33	2.902 (2)	119
C11—H11B···O1^ii^	0.97	2.66	3.134 (2)	111

Symmetry codes:  (ii) −*x*+1/2, *y*+1/2, −*z*+1/2.
